# Infantile Mortality during Childbirth and Its Prevention

**Published:** 1896-07

**Authors:** 


Infantile Mortality During Childbirth and Its Prevention. By A. Brothers, B. S., M. D., Visiting Gynecologist to Beth Israel Hospital, New York ; Attending Gynecologist to the New York Clinic for Diseases of Women ; Instructor in Operative Gynecology at the New York Post-Graduate Medical School and Hospital, etc. Octavo, pp. viii.—179. Price, $1.50. Philadelphia : P. Blakiston, Son & Co., 1012 Walnut street. 1896.

The merit of this work is established by the fact alone that it secured the Jenks prize. The volume is of interest not alone to the pediatrist but to the obstetrician as well. It is divided into twenty-eight chapters, the first four of which consider general facts relating to morbid anatomy, the accoucheur, statistics, and the like ; chapters V. and VI. consider infantile mortality due to maternal causes preceding labor ; chapters VIII. to XX. consider causes for such mortality during labor ; chapters XX. and XXI. fetal causes preceding labor ; chapters XXII. to XXVIII. fetal causes during labor ; chapters XXIX. to XXXVII. fetal causes following labor ; the last chapter gives general considerations on prevention of infantile mortality.

The perusal of this monograph will repay the general practitioner as well as the specialist. L. C. R.

